# Neurobrucellosis: report of 54 cases

**DOI:** 10.1186/s41182-022-00472-x

**Published:** 2022-10-14

**Authors:** HamidReza Naderi, Fereshte Sheybani, Ashkan Parsa, Mahboubeh Haddad, Farzaneh khoroushi

**Affiliations:** 1 Department of Infectious Diseases and Tropical Medicine, Faculty of Medicine, Mashhad University of Medical Sciences, Mashhad, Iran; 2 Faculty of Medicine, Mashhad University of Medical Sciences, Mashhad, Iran; 3 Department of Radiology, Faculty of Medicine, Mashhad University of Medical Sciences, Mashhad, Iran

**Keywords:** Neurobrucellosis, CNS infection, Zoonosis, Meningoencephalitis

## Abstract

**Background:**

Brucellosis is among the most widespread zoonotic diseases worldwide. Although rare, nervous system involvement due to Brucella infection is a major diagnostic challenge in endemic regions.

**Patients and methods:**

This study was a cross-sectional investigation of hospitalized adults with neurobrucellosis from March 2007 to February 2017. We described the clinical characteristics, radiographical and laboratory features, and clinical outcomes of patients with neurobrucellosis.

**Results:**

Fifty-four patients with neurobrucellosis were included. The median age was 35 (interquartile range, 25–50) years, and 32 (59%) cases were male. Thirty-four (63%) patients were stockmen or shepherds. The most common clinical manifestations were fever in 49 (91%) cases, headache in 47 (87%), decreased consciousness in 12 (22%), and seizures in 6 (11%). Meningeal signs were detected in 36 (67%) cases. Brucella species were isolated in five cases from blood or cerebrospinal fluid (CSF). The median of CSF leukocytes was 75 per µL, CSF protein 83 mg/dL, and CSF glucose 39 mg/dL. Only two cases had severe hypoglycorrhachia and one CSF protein ≥ 500 mg/dL. No patient died during hospitalization.

**Conclusions:**

The symptoms of neurobrucellosis could be mild and nonspecific and the classic triad of meningitis is uncommon. Mild CSF pleocytosis of fewer than 50 leukocytes per microliter of CSF was common but severe hyperproteinorrhachia and severe hypoglycorrhachia were rare in neurobrucellosis. Differentiation between neurobrucellosis and systemic brucellosis is important, because more prolonged treatment is indicated for neurobrucellosis, and it could be associated with a broad spectrum of complications that require close follow-up.

## Introduction

Although the incidence of human brucellosis has been reduced to a low level in western European countries and the United States and eradicated in some countries [1, 2], it is among the most widespread zoonotic diseases, being endemic in the Middle East, Mediterranean Europe, Africa and many South American countries [3]. In Iran, human brucellosis remains a huge burden [4], with an increasing trend: brucellosis-related incidence had increased from 88,450 in 2009 to 198,030 in 2015 and, mortality from 244 in 2009 to 578 in 2015 [5]. A variety of factors have been proposed which make the eradication of brucellosis challenging in Iran. Among these factors, some traditional habits such as eating traditionally prepared soft cheese and the use of unpasteurized dairy products by many villagers and also the urban population are among the most important concerns [2].

Nervous system involvement is an infrequent complication of brucellosis that occurs in 3–10% of patients with brucellosis [6–8]. Although globally rare, neurobrucellosis is reported relatively common in countries that are endemic for brucellosis. It has been estimated that neurobrucellosis comprises 0.5% of all episodes of community-acquired CNS infections [9]. Among patients who are admitted due to a variety of complications associated with brucellosis in Iran, 1.4–8% have brucella meningoencephalitis [10–12]. Neurobrucellosis can be classified into three categories including acute meningitis or meningoencephalitis, chronic peripheral form (radiculopathy), and chronic CNS infection (meningoencephalitis, myelitis, cerebellar involvement, cranial nerve palsies) [13].

Diagnosis of neurobrucellosis among laboratory-confirmed cases of brucellosis is confirmed by isolation of brucella from CSF and/or positive anti-Brucella antibodies in CSF [14]. However, diagnosis can be challenging, since serological testing can sometimes yield negative results [15] and the sensitivity of culture-based methods varies depending on laboratory techniques and quantity of bacteria in the CSF [16]. Other diagnostic methods such as CSF metagenomic next-generation sequencing (mNGS) and 16 s rRNA sequencing technique are also used in diagnosing neurobrucellosis [17]; however, they are rarely available in the endemic areas. Although other nonspecific CSF parameters such as CSF oligoclonal bands have also been introduced as rapid and useful diagnostic tools, information regarding the value of these tests in confirmation of diagnosis of neurobrucellosis is limited [18].

Despite a low mortality rate, neurological sequelae remain frequent after neurobrucellosis [19]. It has been estimated that 20–30% of patients with neurobrucellosis developed neurological sequelae [14]. Here, we described the clinical and laboratory characteristics of 54 episodes of neurobrucellosis in adults hospitalized in two main referral centers for CNS infections in Mashhad, Iran.

## Methods

The study was a cross-sectional investigation that was conducted in two main referral centers for CNS infections in Mashhad, Iran. Mashhad is the second largest city in Iran that is located in the northeast of the country. All individuals (≥ 15 years) who were diagnosed with neurobrucellosis between March 2007 to February 2017 were included in the study. Information regarding age, sex, job, history of consumption of unpasteurized dairy products, clinical characteristics, radiographic and laboratory features, and clinical outcome were recorded retrospectively using a checklist.

Brucellosis was diagnosed based on suspected clinical findings and isolation of Brucella species from blood culture and/or positive serum anti-brucella antibodies.

Neurobrucellosis among laboratory-confirmed cases of brucellosis is diagnosed by the presence of any one of the following criteria: (1) suspected symptoms and signs of neurobrucellosis such as severe and persistent headache that interferes with the patients normal activity, insomnia, confusion, depression, behaviour change, incontinence, and neck stiffness, and any neurological finding in examination (2) isolation of brucella from CSF and/or positive anti-Brucalla antibodies in CSF, (3) presence of lymphocytic pleocytosis, elevated protein and decreased glucose levels in CSF, or (4) cranial MRI or CT scan findings [14].

Over the period of the study, conventional culture media were used for CSF culture and standard BACTEC™ Plus Aerobic/F bottles for blood cultures. Conventional culture media were incubated for 14–21 days and BACTEC bottles for 1 week.

According to the Iranian national protocol for diagnosis and management of brucellosis in the endemic areas, the cutoff titre of 1/80 in Wright, 1/40 in Coombs tests*,* and 1/40 in 2ME (2-Mercaptoethanol) test were considered positive, considering other important differential diagnosis and ruling them out. In CSF, a cutoff titre of 1/8 in Wright test was considered positive. Patients with neurobrucellosis were treated with a triple-drug regimen including ceftriaxone, doxycycline, and rifampin for 4 weeks, followed by doxycycline and rifampin for at least 4 months. In those who did not receive or complete a 4-week parenteral ceftriaxone, trimethoprim–sulfamethoxazole was included in the initial regimen as the third agent.

### Statistics

Continuous data were described with median and interquartile ranges and categorical variables with frequency and percentage.

### Research ethics

The ethics committee of Mashhad University of Medical Sciences approved this study with the code of IR.MUMS.MEDICAL.REC.1397.138.

## Results

From March 2007 to February 2017, 54 cases of CNS infections caused by Brucella species were diagnosed. The median age of patients was 35 [interquartile range (IQR), 25–50] years, with an age range of 1–69 years, and 32 (59%) cases were male. Thirty-four (63%) patients were stockmen or shepherds and 51 (94%) mentioned the consumption of unpasteurized dairy products (Table 1).

**Table 1:** Characteristics of patients

Number of cases	54
Age, years	35 [IQR, 25, 50]
Sex, male%	32 (60%)
Stockman	34 (63%)
Consumption of unpasteurized dairy products	51 (94%)
Symptoms and signs	
Fever	49 (91%)
Headache	47 (87%)
Nausea and vomiting	35 (65%)
Sweating	13 (24%)
Altered behavior	8 (15%)
Seizures	6 (11%)
Signs	
Meningeal signs	36 (67)
Focal neurologic deficits	13 (25)
Decreased consciousness	12 (22)
Laboratory data	
CSF leukocytes (/µL)	75 [IQR, 14, 205]
Lymphocyte predominance (%)	84 [IQR, 68, 94]
CSF protein (mg/dL)	83 [IQR, 43, 145]
CSF glucose (mg/dL)	39 [IQR, 24, 56]
ESR, mm/hour	11 [IQR, 6, 20]
CRP, mg/dL	5 [IQR, 0, 32]
Serologic and microbiological tests	
Serum Wright	1/1320 [IQR, 1/160, 1/640]
Serum 2ME-Wright	1/1320 [IQR, 1/160, 1/640]
Serum Coombs-Wright	1/320 [IQR, 1/232, 1/500]
CSF Wright	1/4 [IQR, 1/2, 1/6]
Positive blood culture	3 (5)
Positive CSF culture	2 (4)
Abnormal neuroimaging findings* (*n* = 49)	6 (13)
Mortality	0

*IQR* interquartile range, *CSF* cerebrospinal fluid, *CRP* C-reactive protein, *ESR* erythrocyte sedimentation rate, *2ME* 2-Mercaptoethanol

*Abnormal neuroimaging findings included meningeal enhancement, ischemic foci in frontal lobe, frontal and temporoparietal high signal foci in T2/FLAIR (brain MRI), and hypodense lesions in cerebellum (brain CT scan)

The most common clinical manifestation was fever reported by 49 patients (91%), followed by headache in 47 (87%), nausea and vomiting in 35 (65%), decreased consciousness in 12 (22%), seizures in 6 (11%), and behaviour changes in 8 (5%). Meningeal signs were positive in 36 (67%) cases, focal neurologic deficits in 7 (13%), and papilledema in 6 (11%). Neurologic deficits included cranial nerve (CN) III palsy, bilateral CN VI palsy, combined CN III and VI palsy, and sensorineural hearing loss (CN VIII), each in one. One patient had left hemiparesis, two had decreased visual acuity, and one experienced total blindness. A classic triad of meningitis was found in seven (13%) cases.

Brucella species were isolated in four cases, including two cases with positive blood cultures, one with positive CSF culture, and one with both blood and CSF cultures (Table 2). The organism was isolated within 96 h in all patients who had positive blood cultures. The median of serum Wright, serum 2ME-Wright, serum Coombs-Wright, and CSF Wright were 1/320 [IQR, 1/60–1/640], 1/320 [IQR, 1/60–1/640], 1/320 [IQR, 1/232–1/500], and 1/4 [IQR, 1/2–1/6], respectively.

**Table 2:** Frequency of abnormal CSF and neuroimaging findings in patients with neurobrucellosis

	*n* (%)
Isolation of brucella from CSF and/or positive anti-Brucella antibodies in CSF	15 (28)
Isolation of brucella from CSF	2 (4)
Positive anti-Brucella antibodies in CSF	13 (24)
Presence of lymphocytic pleocytosis, elevated protein and decreased glucose levels in CSF	50 (92)
Lymphocytic pleocytosis in CSF	44 (82)
Elevated protein in CSF	39 (72)
Decreased glucose levels in CSF	28 (52)
Cranial MRI or CT scan findings	6 (11)

CSF: cerebrospinal fluid; MRI: magnetic resonance imaging; CT: computed tomography

The median of CSF leukocytes was 75 [IQR, 14–205] per µL and 24 (44%) cases had less than 50 CSF leukocytes per µL. Nine (17%) cases had CSF leukocytes ≤ 5/µL. The predominance of polymorphonuclear cells was found in 6 (11%) cases. The median of CSF protein and glucose were 83 [IQR, 43–145] mg/dL and 39 [IQR, 24–56] mg/dL, respectively. Only two cases had severe hypoglycorrhachia, defined as CSF glucose ≤ 10 mg/dL and one, CSF protein ≥ 500 mg/dL.

**Fig. 1:**
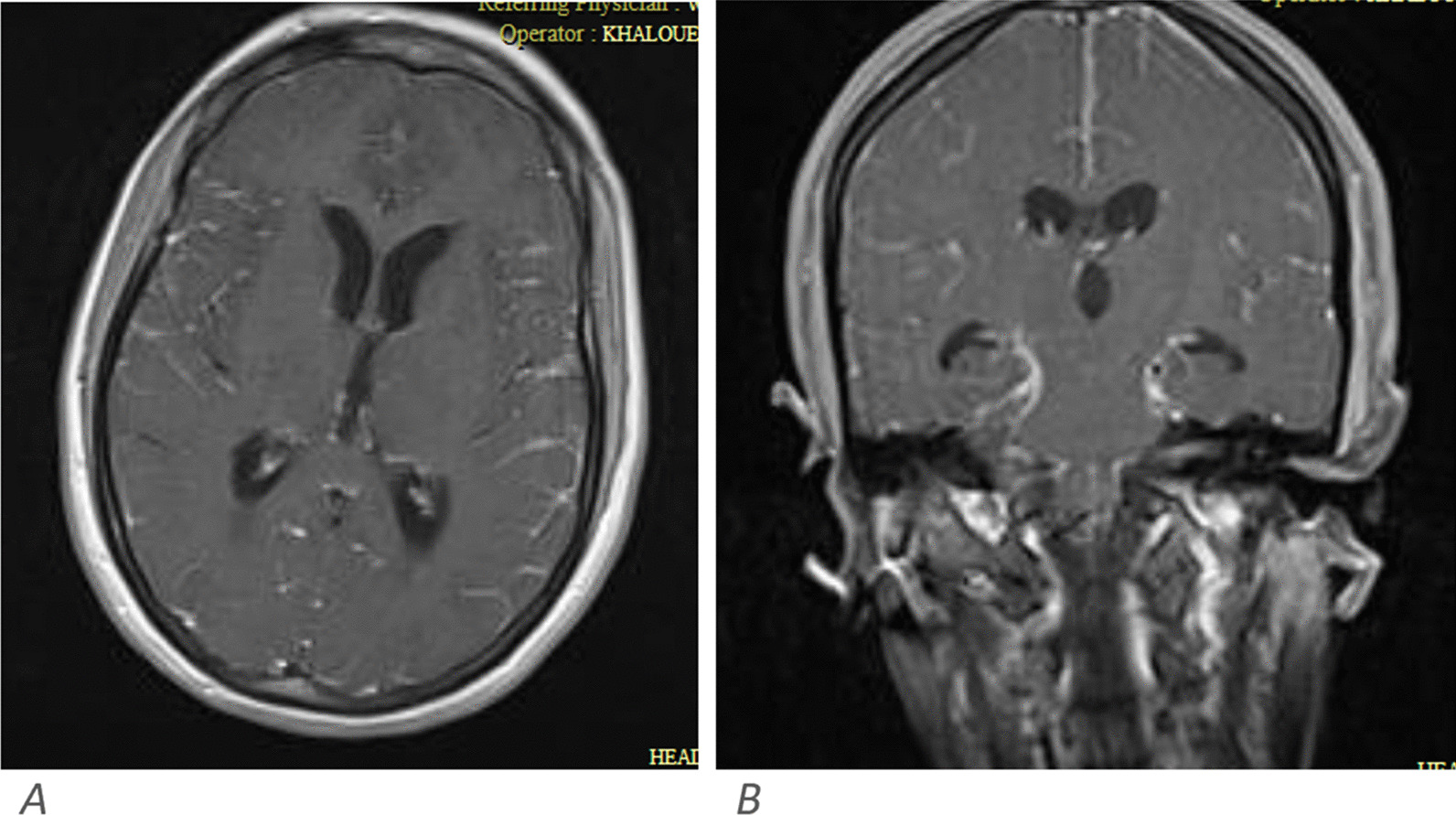
Axial (**A**) and coronal (**B**) post contrast T1-weighted images show diffuse leptomeningeal enhancement

**Fig. 2:**
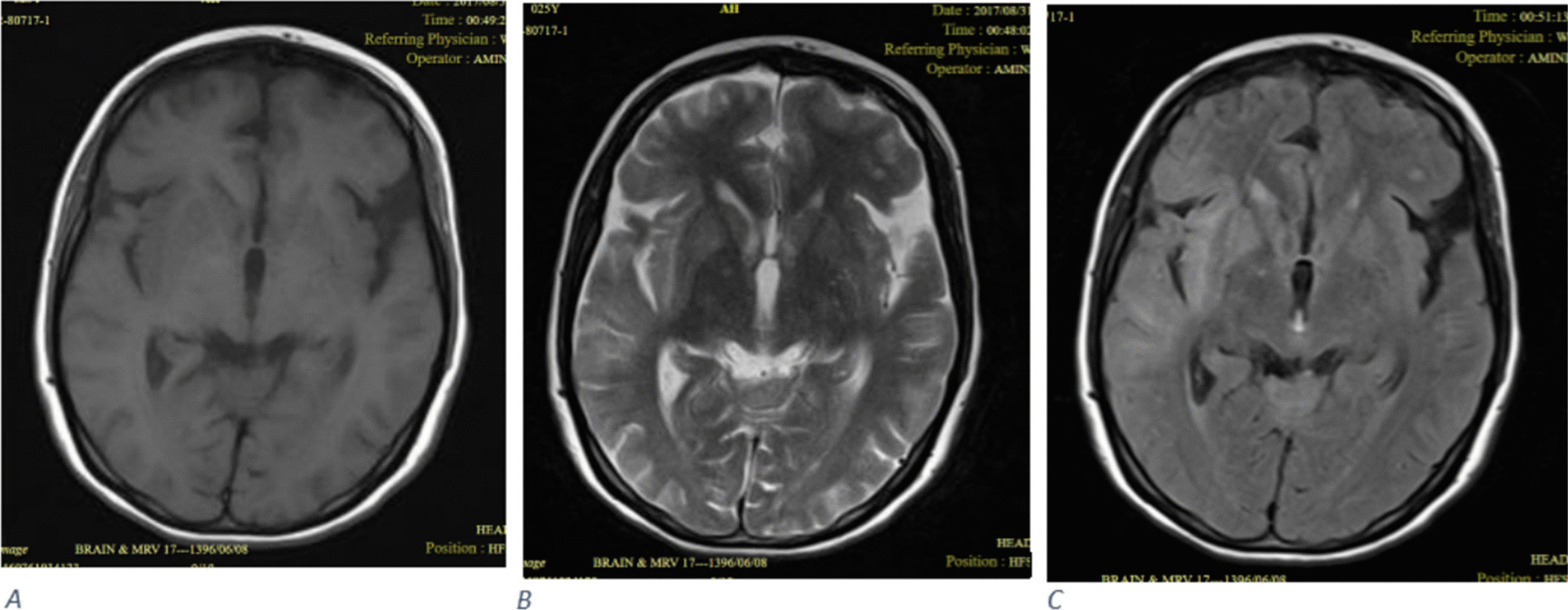
**A** T1-weighted image shows low signal intensity involving right frontotemporal lobe. **B** T2-weighted image demonstrated increased signal intensity in the right frontotemporal lobe. **C** FLAIR image demonstrated increased signal intensity in the right frontotemporal lobe

Neuroimaging studies including brain CT and/or MRI were performed in 47 cases of whom 6 (13%) showed abnormalities. Abnormal neuroimaging findings included meningeal enhancement on post-contrast T1-weighted MR images (Fig. 1), ischemic foci in the frontal lobe on diffusion-weighted MR images, frontal and temporoparietal high signal foci on T2/FLAIR MR images (Fig. 2), and hypodense lesions in cerebellum on brain CT scan.

All patients survived at the time they were discharged from the hospital. However, one patient developed total blindness, one with pseudotumor cerebri-like presentation required CSF shunt insertion, one patient with sensory-neural hearing loss and one with left hemiparesis.

## Discussion

To the best of our knowledge, this is the largest report of patients with neurobrucellosis in Iran and one of the largest worldwide. Our study showed that the most common symptoms of neurobrucellosis are fever, headache, and nausea and vomiting, and the most frequent signs were meningismus and cranial nerve palsies. The classic triad of meningitis was uncommon and occurred in only 13% of patients with neurobrucellosis. Therefore, mild and nonspecific symptoms of Brucella meningitis might be attributed to systemic brucellosis rather than CNS involvement. Nevertheless, the differentiation between systemic brucellosis and neurobrucellosis is important, because more prolonged treatment is indicated for the latter [20]. Furthermore, neurobrucellosis might be associated with a wide spectrum of morbidities [8, 21] that requires close monitoring of neurologic status.

Altered behavior and seizures, although less frequent, occur in 15% and 11% of patients, respectively. Behavior changes within the last month of the presentation were even more frequent (60%) in a previous report of 48 cases of neurobrucellosis in Turkey [14], as compared to our study. These changes are usually mild but patients can also present with frank psychosis [22–24]. Therefore, any altered behavior in a febrile person with epidemiological factors for brucellosis could be suggestive of neurobrucellosis and should be evaluated further.

About half of our patients with neurobrucellosis had mild CSF pleocytosis of fewer than 50 leukocytes per microliter of CSF, including 17% with five or fewer leukocytes per microliter. Previous studies showed that in cases with CSF leukocytes fewer than 50 per microliter, non-infectious neurological disorders should be considered as the main differential diagnoses [25]; however, neurobrucellosis is another important diagnosis that should be considered in those with compatible clinical syndromes and predisposing epidemiological factors. Although lymphocytic pleocytosis was a prominent feature occurring in 90%, about one in ten cases had CSF polymorphonuclear cell predominance.

The yield of Brucella culture from CSF of patients with neurobrucellosis is low (5–30%) [6] and most cases with neurobrucellosis are diagnosed by serological methods [14]. In our study, only five episodes of neurobrucellosis (9%) were confirmed by culture-based blood and/or CSF microbiological tests. Previously, another case series that provided information regarding culture-confirmed cases of neurobrucellosis from Iran reported positive blood or CSF culture in 5 out of 31 episodes, all of which were caused by *B. melitensis* [26].

Neuroimaging findings in neurobrucellosis are variable and can resemble other infectious or inflammatory conditions. Four patterns of CNS involvement on imaging have been described in patients with neurobrucellosis: normal, inflammation, white matter changes and vascular changes. Inflammation is recognized by granulomas, or meningeal, perivascular space, or lumbar nerve roots enhancement [27]. Vascular involvement can also be seen, resulting in lacunar infarcts, small haemorrhages or venous thrombosis [28].

One of the most important differentials for neurobrucellosis in TB endemic areas is neurotuberculosis [29]. In fact, in the countries which are endemic to TB and brucellosis, differentiation between neurotuberculosis and neurobrucellosis is very challenging. Several criteria such as Thwaites and Lancet scoring systems have been introduced for the rapid diagnosis of TB meningitis. However, they can misdiagnose neurobrucellosis as neurotuberculosis [30]. Clinically, hearing loss due to vestibulocochlear nerve involvement has been reported as a frequent and unique feature of neurobrucellosis in differentiation from neurotuberculosis [14]. Accordingly, electrophysiological studies have been suggested to detect subclinical vestibulocochlear nerve involvement, as it may assist in pointing toward the diagnosis of neurobrucellosis [29]. Our study showed CSF protein > 500 mg/dL and severe hypoglycorrhachia were uncommon among cases of neurobrucellosis. Therefore, when a very high CSF protein level is among the known features of neurotuberculosis, it is uncommon in neurobrucellosis and should raise the possibility of neurotuberculosis or other differentials as the most probable diagnosis.

## Conclusions

Neurobrucellosis is a diagnostic challenge in Brucella endemic areas. The symptoms could be mild and nonspecific and the classic triad of meningitis is uncommon. Behavior changes are among the important clinical features are neurobrucellosis. Differentiation between neurobrucellosis and systemic brucellosis is important, because more prolonged treatment is indicated for neurobrucellosis and it could be associated with a wide spectrum of complications that require close neurologic monitoring.

Mild CSF pleocytosis of fewer than 50 leukocytes per microliter of CSF was common and identified in about half of the cases of neurobrucellosis. Nevertheless, severe hyperproteinorrhachia and severe hypoglycorrhachia were rare in neurobrucellosis. The yield of Brucella culture from CSF and blood is low in cases of neurobrucellosis and the culture-confirmed diagnosis was made in less than 10% of patients.

An important differential for neurobrucellosis in TB endemic areas is neurotuberculosis**.** When a very high CSF protein level is among the known features of neurotuberculosis, it is rare in neurobrucellosis and should raise the possibility of neurotuberculosis as a more probable diagnosis.

## Data Availability

The data sets generated during the current study are available from the corresponding author on reasonable request.
